# Birds Perceive More Intraspecific Color Variation in Bird-Pollinated Than Bee-Pollinated Flowers

**DOI:** 10.3389/fpls.2020.590347

**Published:** 2020-11-17

**Authors:** Kenneth D. Whitney, Asher K. Smith, Thomas E. White, Charles F. Williams

**Affiliations:** ^1^Department of Biology, University of New Mexico, Albuquerque, NM, United States; ^2^Rocky Mountain Biological Laboratory, Crested Butte, CO, United States; ^3^School of Life and Environmental Sciences, The University of Sydney, Sydney, NSW, Australia; ^4^Department of Biological Sciences, Idaho State University, Pocatello, ID, United States; ^5^Ray J. Davis Herbarium, Idaho Museum of Natural History, Pocatello, ID, United States

**Keywords:** plant–pollinator interactions, signaling, reflectance spectra, bee vision, avian vision, natural selection, color polymorphism, color saturation

## Abstract

Pollinator-mediated selection is expected to constrain floral color variation within plant populations. Here, we test for patterns of constraint on floral color variation in 38 bee- and/or hummingbird-pollinated plant species from Colorado, United States. We collected reflectance spectra for at least 15 individuals in each of 1–3 populations of each species (total 78 populations) and modeled perceived color variation in both bee and bird visual spaces. We hypothesized that bees would perceive less intraspecific color variation in bee-pollinated species (vs. bird-pollinated species), and reciprocally, birds would perceive less color variation in bird-pollinated species (vs. bee-pollinated species). In keeping with the higher dimensionality of the bird visual system, birds typically perceived much more color variation than bees, regardless of plant pollination system. Contrary to our hypothesis, bees perceived equal color variation within plant species from the two pollination systems, and birds perceived more color variation in species that they pollinate than in bee-pollinated species. We propose hypotheses to account for the results, including reduced long-wavelength sensitivity in bees (vs. birds), and the ideas that potential categorical color vision in birds and larger cognitive capacities of birds (vs. bees) reduces their potential discrimination against floral color variants in species that they pollinate, resulting in less stabilizing selection on color within bird-pollinated vs. bee-pollinated species.

## Introduction

Among other traits such as scent and size, flower color is a major signal used by pollinators to identify and choose their host plants (Fenster et al., 2004; Dyer et al., 2012; Schiestl and Johnson, 2013). Because foraging decisions affect visitation, pollination, pollen export and seed set – and thus plant fitness – pollinators can exert selection on flower color (Waser and Price, 1981; Rausher, 2008; Renoult et al., 2013; Caruso et al., 2019). When flower color variants arise via mutation within a plant population, they should be frequently selected against, as pollinators can exhibit positive frequency dependence in their floral color choices (Smithson, 2001; Eckhart et al., 2006). Thus, a general prediction is that pollinator-driven stabilizing selection should limit intrapopulation variation in floral color (Waser and Price, 1983; Fenster et al., 2004; Rausher, 2008).

Importantly, however, clade-specific color-sensitive receptors and cognitive mechanisms (Renoult et al., 2017) mean that floral color variation is in the eye (and brain) of the beholder. Thus, pollinator-imposed constraints may only be obvious within the bounds of the visual space of the pollinator, and color variation may be less constrained in the visual spaces of non-pollinators (Paine et al., 2019). Central to testing hypotheses about constraints on flower color is the idea of discrimination thresholds within pollinator visual spaces. Within a visual space, greater distance between a pair of colors predicts greater discriminability, but all organisms have thresholds below which discrimination is not possible (e.g., Wyszecki and Stiles, 2000; Dyer and Chittka, 2004; Olsson et al., 2018). For example, within the color-hexagon model of bee vision (Chittka, 1992), bees are typically unable to distinguish colors separated by a Euclidean distance of 0.11 units (e.g., Dyer and Chittka, 2004). Taxon-specific discrimination thresholds allow standardized comparisons of the color variation perceived by different animal taxa (viewing the same set of signals) or perceived by a single animal taxon (viewing different sets of signals).

Using these methods, patterns of floral color variation were recently examined for 34 populations of 14 species of New Mexican bee-pollinated plants, using modeling of visual spaces of bees, birds and humans. For >70% of populations, >95% of pairwise flower–flower comparisons were indistinguishable to bees, consistent with (but not proving) a history of stabilizing selection on flower color mediated by the bee visual system (Paine et al., 2019). Further, these pairs of conspecific flowers were typically visually distinct to humans and birds (non-pollinators of these plants). These findings suggest that human-perceived floral color variation within populations might persist because it is effectively invisible to pollinators. Under these conditions, human-perceived color may evolve neutrally (via drift) or via indirect selection on correlated characters such as drought- or herbivore-resistance, given known pleiotropy between flower pigmentation and these characters (Simms and Bucher, 1996; Schemske and Bierzychudek, 2001; Warren and Mackenzie, 2001; Irwin et al., 2003; Strauss et al., 2004; Vaidya et al., 2018).

Investigations into intraspecific flower color variation are in their infancy (van der Kooi et al., 2019), and specific hypotheses relating perceived intraspecific variation in flower color to the interaction between pollinator visual systems and pollination systems have not yet been developed. While one might be tempted to hypothesize that a pollinator should perceive less color variation within plant species it pollinates, relative to variation perceived by a non-pollinator viewing the same species, this is unlikely to be uniformly true because of differences in overall visual acuity of different animal groups. Dimensionality (the number of receptor types) differs among pollinators. The linear separability of points in any colorspace will generally increase when projected into a higher-dimensional space (Cover, 1965), suggesting that perceived color differences will tend to increase with the number of available input channels. Thus, tetrachromatic birds should typically have finer spectral resolution than trichromatic bees, though taxon-specific variation in receptor sensitivities and post-receptor processing mean that bees can likely achieve finer discrimination than birds in certain regions within the UV-through-green wavelengths (Vorobyev, 1997; Dyer and Chittka, 2004; Caves et al., 2018). These considerations lead us to propose a distinct hypothesis: if pollinator-mediated stabilizing selection has been important in shaping flower color, a pollinator should perceive less color variation within plant species it pollinates, relative to species it does not pollinate (and thus has had no opportunity to shape). In terms of a dataset consisting of flower colors for populations of plant species pollinated by two different pollinator groups, this would manifest as perceived variation being a function of a visual space × pollination system interaction. Here, we test for such a pattern using 78 populations of 38 bee- and/or hummingbird-pollinated plant species from Colorado, United States. We use visual modeling of discrimination thresholds to estimate relative amounts of perceived color variation. Specifically, we hypothesize that bees should perceive less intrapopulation color variation in bee-pollinated (vs. bird-pollinated) plant species; and reciprocally, birds should perceive less intrapopulation color variation in bird-pollinated (vs. bee-pollinated) plant species.

## Materials and Methods

### Study Area

The study was conducted in the Elk Mountains surrounding the Rocky Mountain Biological Laboratory (RMBL) in Gothic, CO, United States (N 38.95807°, W 106.98853°; elev. 2889 m). The area is topographically and biotically diverse (Zorio et al., 2016), with over 1000 species of flowering forbs and shrubs reported from a 10 km radius of RMBL^1^. Bees, flies and hummingbirds (Trochilidae) are the major pollinators in this ecosystem; hummingbirds are the only bird pollinators. Lepidopteran pollinators are present but less common (Miller, 1978; Moldenke and Lincoln, 1979; Campbell et al., 1998). Elevational gradients in flower color in this area have been previously described (Gray et al., 2018).

### Study Species and Field Collection

During Summer 2019 (June 1st – August 12th), we opportunistically collected population-level samples from 78 populations of 38 flowering plant species (1–3 populations per species, mean = 2.1). Table 1 presents the species information. These species generally represented the commonly encountered bee- and bird-pollinated angiosperm forbs and shrubs of the area, but phylogenetic representation was broadened by typically limiting consideration to no more than two species per plant family. However, we targeted bird-pollinated species to increase their representation, since there are fewer bird-pollinated than insect-pollinated species in the area; this resulted in heavier sampling in certain families (e.g., Orobanchaceae; Table 1). In total we sampled from 20 families, with a mean of 1.9 and a range of 1–6 species per family (Table 1). For all species not readily identifiable in the field, one or more voucher specimens were deposited in the RMBL herbarium (specimen list available via soroherbaria.org with Asher K. Smith as collector and “RMBL” as institution).

**TABLE 1:** Plant species examined in this study and their pollination systems.

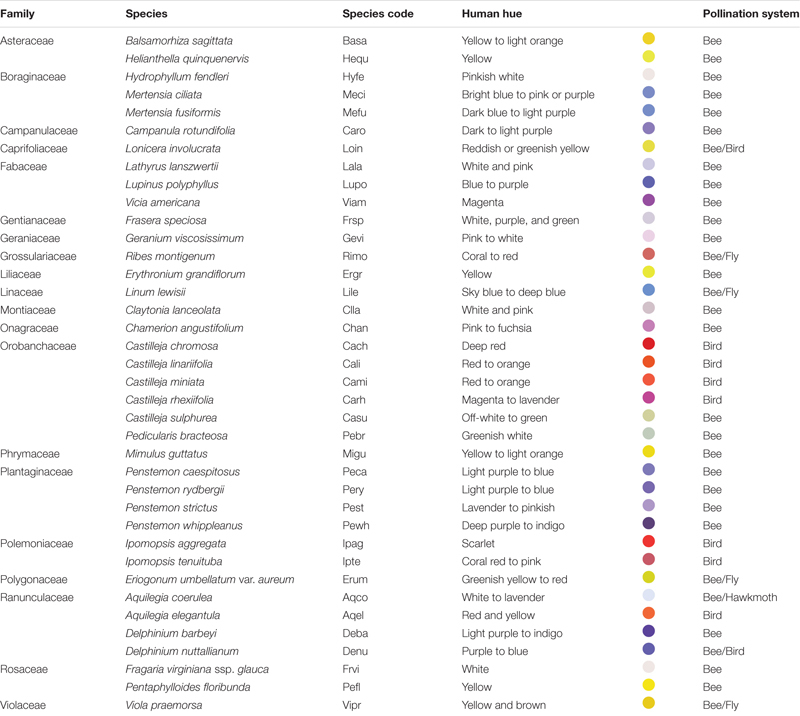

*Colored dots representing the dominant flower hue in human visual space were created by extracting RGB values from a representative pixel from a digital image of each species. Citations for pollination system information are given in Supplementary Appendix Table A2.*

Population-level samples consisted of an individual flower or inflorescence collected from each of 15 individual plants (1239 total individual plants sampled). For the family Asteraceae, an inflorescence is morphologically integrated to function as a single flower, and therefore we treat their inflorescences as “flowers.” Similarly, we collected inflorescences for species for which bracts contribute to showiness and pollinator attraction (e.g., the genus *Castilleja*). We defined populations spatially; we sampled a given species only from locations >1 km distant or at >100 m elevation change from other sampling areas and separated by areas where the species was not present.

### Pollination System Classifications

To identify the pollinators for each of the 38 plant species, we conducted a literature review, supplemented with local natural history knowledge. Categorizations of the pollination system (“Bee,” “Bird,” “Bee/Fly,” “Bee/Bird,” and “Bee/Hawkmoth”) for each species and source citations are presented in Supplementary Appendix Table S2. Single categorizations (e.g., “Bee”) indicate species with a single expected dominant pollinator group, but do not preclude that other pollinator groups contribute in minor roles. Dual categorizations (e.g., “Bee/Fly”) denote a mixed pollination system where either there is no dominant group (e.g., ∼50:50 contributions by two groups), or that there is not enough resolution to identify the dominant group.

### Spectrophotometry

We took spectral readings within 12 h of flower collection. For species with multiple human-distinguishable color patches present within a single flower or inflorescence, we chose a patch representing the greatest surface area when considered from the viewpoint of an approaching pollinator. Typically, an individual petal or bract was mounted on cellophane tape and affixed to the bottom of the probe-holder block. The same petal or bract region was used consistently across all individuals within a species (for details on flower preparation for each species, see Supplementary Appendix Table S3). Spectrometer readings (spanning 300–700 nm) were taken using an Avantes model 2048 spectrometer, a bifurcated coaxial fiber optic reflectance probe (Avantes FCR-7uv200-2-ME) and an AvaLight-XE xenon light source (Avantes BV, Apeldoorn, Netherlands). Calibration was first made relative to a diffuse white PFTE tile (Avantes WS-2). Integration time was 10 ms. To reduce specular reflectance (Chittka and Kevan, 2005), measurements were taken with the fiber optic probe held at 45° to, and at 8.0 mm from, the flower surface, with the petal tip facing away from the probe. Our sampling design of one spectrum per flower was informed by Dalrymple et al. (2015), which indicated that flower color can be quite precisely estimated with a single measurement.

### Spectral Processing and Visual Modeling

We used the R package ‘pavo’ (Maia et al., 2019) for spectral processing and visual modeling. We first trimmed the spectra to 300–700 nm and set spurious negative reflectance values to zero. We then estimated the subjective perception of floral signals using the receptor-noise limited model for birds (Vorobyev and Osorio, 1998) and the color hexagon model for bees (Chittka, 1992). Each model allows colors to be represented as points in a space delimited by the number and sensitivity of photoreceptors, while accounting for factors such as veiling and incident light, the structure of viewing backgrounds and signals, and more species-specific features of visual processing and perception (Kemp et al., 2015; Maia and White, 2018).

In these color spaces, the distances between points can be interpreted as measures of the subjective difference between colors, with values less than a behaviorally validated ‘threshold’ of discrimination likely to be indiscriminable to a given viewer. In the receptor-noise limited model for birds, color distances are expressed as weighted Euclidean distances (ΔS), with a value of 1.0 for diurnal birds taken to (conservatively) delimit the threshold below which colors are expected to be indiscriminable under ecologically relevant conditions (reviewed in Wyszecki and Stiles, 2000; Olsson et al., 2018). In the color hexagon for bees, hue is indicated by the radial angle and saturation (spectral purity) is indicated by the distance from the (0,0) origin (Chittka, 1992). Testing of bumblebee and honeybee behavior under laboratory conditions has determined that colors separated by a Euclidean distance of 0.11 ‘hexagon units’ are indiscriminable without aversive differential conditioning, i.e., training with simultaneously presented rewarding and aversive colored stimuli (Dyer and Chittka, 2004; Dyer and Neumeyer, 2005; Dyer, 2006). We used the receptor sensitivities of *Apis mellifera* (Peitsch et al., 1992) as a representative bee pollinator, since the hexagon model is well validated in this species and the sensitivities of photopigments underlying trichromatic vision in the Hymenoptera are highly conserved (Briscoe and Chittka, 2001).

For birds, we used the visual phenotype of an average violet-sensitive (VS) avian viewer for receptor-noise modeling, as the preponderance of evidence suggests that hummingbirds have a VS rather than UVS (ultraviolet-sensitive) system (reviewed in Stoddard et al., 2020). To test the robustness of our results to this assumption, we also modeled birds as UVS; doing so did not qualitatively change any of the patterns or significance levels (results not shown). We specified a relative receptor density of 1:2:2:4 (ultraviolet: short: medium: long wavelength receptors), used a signal-to-noise ratio yielding a Weber fraction of 0.1 and a D65 ‘standard daylight’ illuminant, and assumed that noise is proportional to the Weber fraction and independent of the magnitude of receptor stimulation (Vorobyev and Osorio, 1998).

### Statistical Analysis

#### Background

To explore spectral differences between plants exhibiting different pollination systems, we examined saturation (spectral purity). Bees are expected to have selected for highly saturated colors (Lunau, 1990; Lunau et al., 1996; Rohde et al., 2013). We fit linear mixed-effects models to the data via maximum likelihood using R package ‘lme4’ (Bates et al., 2015), with saturation as the response variable, and visual system (bee vs. bird), pollination system, and their interaction as predictors. Plant species and population (nested within species) were included as random effects. This analysis focused on only plant species with “Bird” and “Bee” pollination systems (*n* = 62 populations of 31 species).

#### Bee and Bird Perception of Intrapopulation Floral Color Variation

To address our focal question, we compared pairwise distances in color space to the relevant discrimination threshold, as delineated above (see also Paine et al., 2019). With 15 samples, there are 15!/(2!(15-2)!) = 105 possible pairwise (flower–flower) comparisons per population. The fraction of these intrapopulation comparisons that are discriminable to a given viewer we call the ‘fraction discriminable.’ We tabulated comparisons using a custom R script (R Core Team, 2020).

To compare levels of variation perceived by bees vs. birds, we fit linear mixed-effects models to the data via maximum likelihood using R package ‘lme4’ (Bates et al., 2015). Fraction discriminable was the response variable, with visual system (bee vs. bird), pollination system, and their interaction as predictors. Plant species and population (nested within species) were included as random effects. The main analysis focused on only plant species with “Bird” and “Bee” pollination systems (*n* = 62 populations of 31 species). To test the robustness of the results to inclusion of the seven species with mixed pollination systems in various ways, separate models were examined where (1) the two “Bee/Bird” species were included with “Bee,” for a total of 33 species; (2) the two “Bee/Bird” species were included with “Bird,” for a total of 33 species; and (3) the four “Bee/Fly” and the single “Bee/Hawkmoth” species were both included with “Bee,” for a total of 36 species.

To examine whether the amount of floral color variation is correlated across visual spaces, we regressed bird fraction discriminable on bee fraction discriminable. Alternative models with and without two additional predictors, a) pollination system (bee vs. bird) and b) the interaction between pollination system and visual system, were evaluated using AICc. For visualization we used package ‘visreg’ (Breheny and Burchett, 2017).

## Results

### Background

Overall, we examined 24 bee-pollinated species, seven bird-pollinated species, and seven species with mixed pollination systems (four “Bee/Fly,” one “Bee/Hawkmoth” and two “Bee/Bird”); for reflectance spectra, see Supplementary Appendix Figure S1. Colors of bee-pollinated species were more highly saturated than those of bird-pollinated species in bee visual space (*p* < 0.0001), as expected, but birds do not perceive differences in saturation between flowers of the two pollination systems (*p* = 0.12, Supplementary Appendix Figure S2).

### Bee and Bird Perception of Intrapopulation Floral Color Variation

**FIGURE 1:**
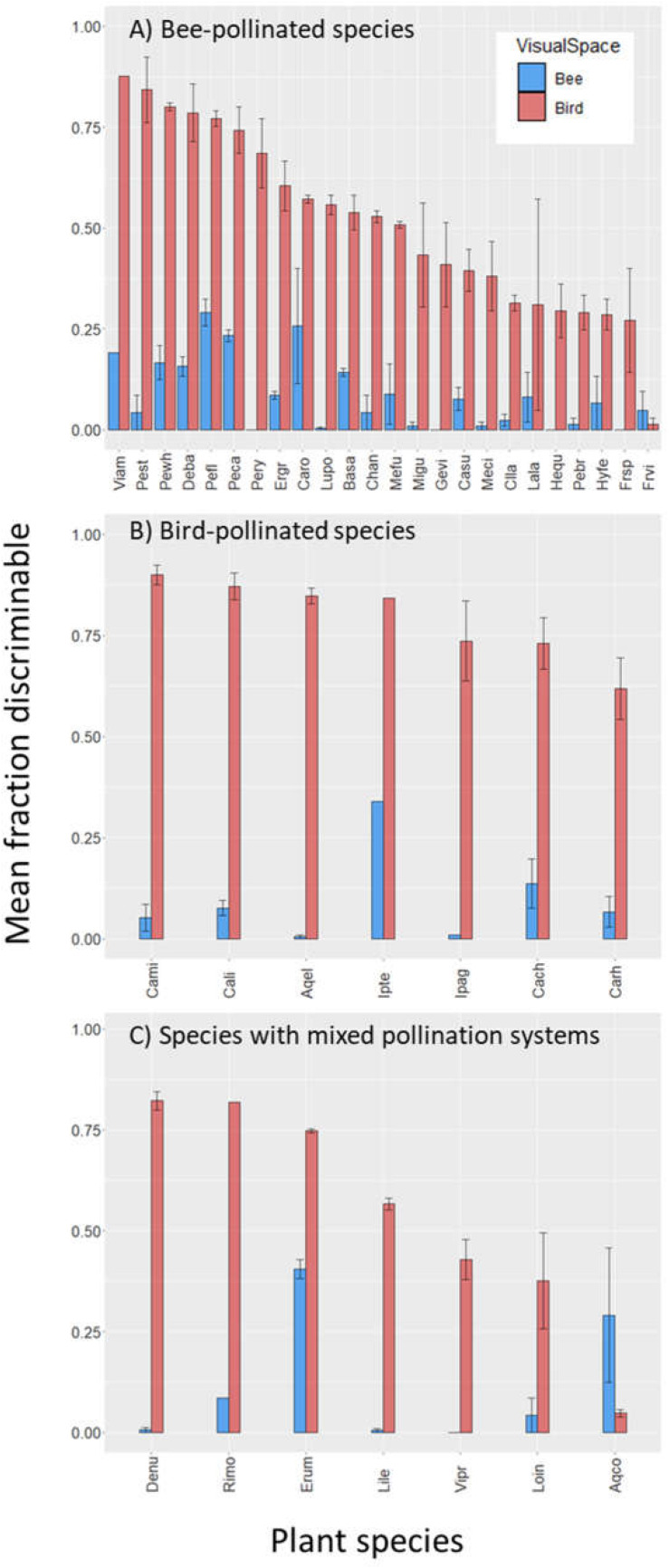
Intrapopulation flower color variation as perceived by bees and birds for 38 species of Rocky Mountain plants. Bars represent the average fraction ± SE (across 1–3 populations per species) of intrapopulation flower–flower comparisons that are discriminable to each viewer. Standard error bars are not present for plant species represented by a single population. Species are presented on the *x*-axis in order of decreasing variation within bird visual space; names associated with the species codes are given in Table 1. **(A)** Bee-pollinated species; **(B)** bird-pollinated species; **(C)** species with mixed pollination systems: “Bee/Fly” (Rimo, Lile, Erum, Vipr), “Bee/Hawkmoth” (Acqo), or “Bee/Bird” (Denu, Loin).

Across all 38 plant species, mean intrapopulation floral color variation (percent flower pairs discriminable) was 8.6% (range 0–45.7%) within bee visual space, and 56.2% (range 0–92.4%) within bird visual space (Figure 1). Across plant populations, bee-perceived and bird-perceived variation was positively correlated (Supplementary Appendix Figure S3, *p* = 0.0003, adj. *r*^2^ = 0.38).

**FIGURE 2:**
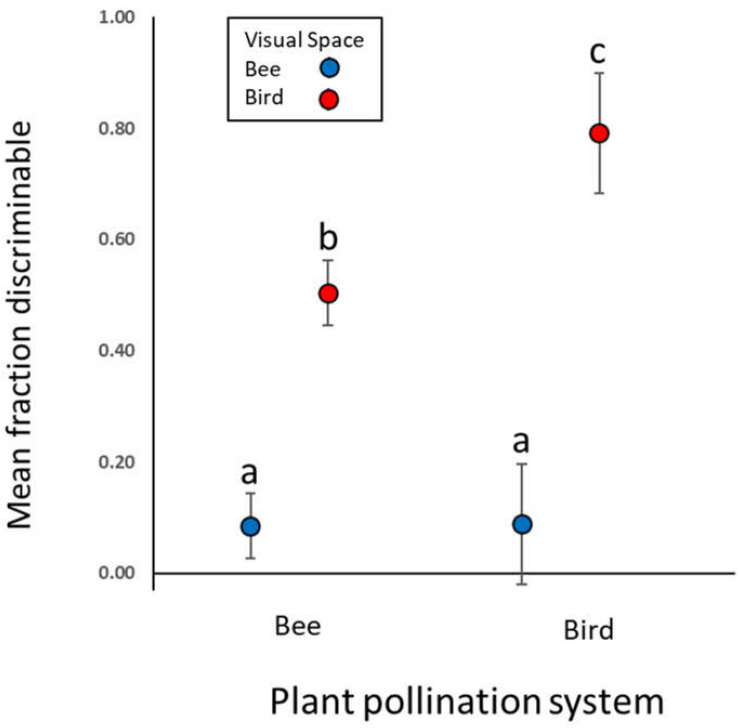
Mean intrapopulation flower color variation in bee and bird visual spaces. Points represent estimated model means (with 95% CI) for the fraction of intrapopulation flower–flower comparisons that are discriminable to each viewer. Data are for 62 populations of 31 Rocky Mountain plant species, grouped by pollination system (24 bee-pollinated and seven bird-pollinated species). Means not sharing a letter differ significantly at *p* < 0.05.

In the main analysis including only the 24 “Bee” and seven “Bird” species, birds perceive greater variation than bees among flowers of both bee- and bird-pollinated plant species (Figure 2; main effect of visual system χ^2^ = 488.6, *p* < 0.0001). With regard to our main hypothesis, we did detect the expected visual space × pollination system interaction (χ^2^ = 30.2, *p* < 0.0001). However, the patterns ran contrary to the hypothesis: bees did not perceive less color variation in bee-pollinated than bird-pollinated species (instead perceiving equal variation in the two groups, Figure 2, contrast t ratio = −0.052, *p* = 0.9585); nor did birds perceive less variation in bird-pollinated than bee-pollinated species (instead, birds perceived more variation in species they pollinate, Figure 2, contrast t ratio = −4.695, *p* < 0.0001). As a consequence of this large difference in bird visual space, averaged across visual spaces, bee-pollinated species exhibited overall lower perceived floral color variation than did bird-pollinated species (main effect of pollination system, χ^2^ = 7.4, *p* = 0.0065).

The above patterns were qualitatively similar (all effects remained significant and in the same directions) when the two “Bee/Bird” species (*Delphinium nuttallianum*, *Lonicera involucrata*) were included with “Bee” species, when they were instead included with “Bird” species, or when “Bee/Fly” and “Bee/Hawkmoth” species were both included with “Bee” species (Supplementary Appendix Table S4).

## Discussion

Our exploration of intrapopulation flower color variation as perceived by bee and bird pollinators found two major patterns. First, birds routinely perceive more intrapopulation variation than do bees; across the plant species examined here, birds were able to distinguish ∼6–9 times more variation than bees (for bee-pollinated and bird-pollinated species, respectively). Second, the evidence is not consistent with the hypothesis that a pollinator should perceive less variation within plant species it pollinates, relative to species it does not pollinate. We estimated that bees perceived equal color variation within populations of bee-pollinated (vs. bird-pollinated) species; further, and in a striking departure from the hypothesis, birds perceived *more* flower color variation within plant species for which they are the dominant pollinator, relative to bee-pollinated plant species.

### Bee Perception of Intrapopulation Flower Color Variation

We found that bees typically perceive very little color variation within plant populations that they visit, even for species that exhibit clear variation to humans. These results echo those of Paine et al. (2019), who examined a non-overlapping set of bee-pollinated plant species roughly 450 km to the south of the current study area. However, the inclusion of bird-pollinated species in the current study made it clear that low bee-perceived variation holds for plant species for whom bees play little or no role in pollination. This pattern may arise if the signals of non-bee pollinated species are constrained by their own pollinators (with the shared fundamentals of color vision translating the ‘signature’ of low-variation to bee visual space), although we note this is unlikely in the current study given the large amount of spectral variation found in populations of bird-pollinated species. Alternatively, the pattern could arise if variation within non-bee pollinated species is biased toward a spectral region to which bees are less sensitive. This scenario is particularly plausible in the current study given that our non-bee pollinators are tetrachromatic birds which possess a richer color-sense that extends into the long-wavelength ‘red’ region (Chittka, 1992; Hart, 2001; Stoddard et al., 2020). The signals of all seven species classified as bird-pollinated in this study are dominated by long-wavelength reflectance (i.e., they are ‘red,’ in human-subjective terms; Table 1), as consistent with general evidence of partitioning between insect and bird-pollinated flowers along a ‘red arm’ (Burd et al., 2014). Thus any variation within the ‘red arm’ of our sampled bird-pollinated species will be relatively difficult for bee viewers to perceive, meshing with a theme in the literature that long-wavelength reflection may be part of a suite of adaptations making it difficult for bees to find and visit bird flowers (Castellanos et al., 2004; Wessinger et al., 2014; Bergamo et al., 2016; see also Chittka and Waser, 1997). Further, with the exception of *Aquilegia elegantula*, none of our seven red bird-pollinated species reflect in the UV (Supplementary Appendix Figure S1, panel A). This pattern matches expectations that hummingbird-pollinated red flowers should lack UV reflectance; such flowers are achromatic in bee visual space and difficult for bees to detect against the background, perhaps allowing these floral signals to occupy a ‘private niche’ for hummingbirds (Lunau et al., 2011). A final general possibility is that low perceived variation may result from forces other than selection by the pollinator group in question. Given connections between the biosynthetic pathways of pigments and other important compounds in plants, flower color can be under indirect selection from many different biotic and abiotic selective agents (Strauss and Whittall, 2006; Caruso et al., 2019).

### Bird Perception of Intrapopulation Flower Color Variation

As expected, birds perceived larger amounts of intrapopulation floral color variation than did bees, across the dataset. The simple difference in dimensionality of the two visual spaces will on-balance give tetrachromatic birds finer spectral resolution than trichromatic bees, as generally expected (Cover, 1965; Vorobyev, 1997). In addition, spectral filters, such as oil droplets, are ubiquitous among birds and serve to minimize the overlap in sensitivity between receptor types (Vorobyev, 2003; Hart and Vorobyev, 2005). Such filters enhance discrimination as compared to bee pollinators, whose receptors instead retain the broad-band sensitivity inherent to visual pigments (Peitsch et al., 1992). Thus our finding that birds likely perceive greater intraspecific floral variation than bees, irrespective of plant pollination system, is consistent with these general differences in their visual systems.

In contrast to our hypothesis that a pollinator should perceive less variation within plant species it pollinates, relative to species it does not pollinate, birds were estimated to perceive *more* variation within bird-pollinated relative to bee-pollinated flowers. This pattern is unlikely to arise simply because (as argued above) birds have an overall richer color-sense than bees. Because birds sample the full visible spectrum relatively efficiently (Vorobyev, 1997), we infer that our bird-pollinated species are indeed more spectrally variable in an absolute sense. We propose several hypotheses to account for this increased variation and the resulting discrepancy between the data and our hypothesis. They share the common theme that birds may not generate as strong selection against color variants as do bees, and thus ‘tolerate’ higher flower color variation than bees within the plant species that they pollinate.

First, recent research raises the intriguing possibility that categorical color perception may be common among birds both in signaling and non-signaling contexts (Caves et al., 2018; Zipple et al., 2019), and so may shape the functional relevance of apparent signal variation. Categorical perception suggests that (at least some) birds group color stimuli into categories, most likely during post-retinal processing, and canalize a consistent response to those stimuli which share a category despite possessing the low-level sensory apparatus to allow for discrimination (Caves et al., 2018; Zipple et al., 2019). A parallel in human vision is humans’ categorical perception of discrete bands in a rainbow, despite the continuously varying wavelengths involved. In examining zebra finch (*Taeniopygia guttata*) vision in the context of mate choice and foraging, boundaries within the orange-red (Caves et al., 2018) and blue-green (Zipple et al., 2019) regions of the spectrum have been documented, such that color pairs on one side of the boundary were less readily distinguished than pairs which spanned it, despite approximately equal color distances between all pairs. For example, in a foraging experiment where food was available under bicolored (but not unicolored) disks, finches were less able to increase foraging success by targeting bicolored disks when the two colors were on one side of the orange-red boundary, than when the two colors spanned the boundary, despite equal discriminability (Caves et al., 2018). As applied to floral signals, then, these results suggest that heightened color variation in bird visual space is not necessarily actionable or functionally relevant, and so (compared to bees) may not as often result in differential fitness among floral color variants.

A related, but more general, possibility is that the larger cognitive capacities of birds (vs. bees) could reduce their potential discrimination against floral color variants in species that they pollinate, resulting in less stabilizing selection on color within the avian visual space than within the bee visual space. In short, the capacities of the avian brain may mean that while birds may perceive greater variation in flower color (as outlined above), they are subsequently able to recognize, classify, and/or remember varying signals as equivalent food resources; thus their foraging decisions may not punish color variants to the extent that bees’ might. While bees seem to maintain some level of innate color preference even after accumulating foraging experience (Smithson and Macnair, 1996; Rohde et al., 2013), hummingbirds can be easily trained to switch their color preferences from red to white flowers if the rewards are better (Meléndez-Ackerman et al., 1997). The color signals of bird-pollinated flowers may therefore be (comparatively) less constrained because, unlike bees, such variation is contended with during higher-level processing.

Finally, different pollinating animals emphasize different cues to make their foraging decisions. It may be that color cues are less important to birds than to bees, and thus are subject to less stabilizing selection by the former. Both hummingbirds and bees are known to use olfactory cues to select flowers for visitation (Kessler and Baldwin, 2007; Byers et al., 2014). In a test with *Mimulus* hybrids, pigmentation had a weaker effect on determining hummingbird visitation than bee visitation, with nectar volume (perhaps signaled via scent) serving as a better predictor for hummingbird visitation rates (Schemske and Bradshaw, 1999).

### Implications for Floral Evolution

It is possible, though by no means guaranteed, that the apparent high tolerance of flower color variation by bird pollinators means that bird-pollinated lineages could have higher standing genetic variation for flower color (vs. bee-pollinated lineages). Given increasing knowledge about the genetic basis of flower color (e.g., Streisfeld and Rausher, 2009), this hypothesis could be tested. If present, higher standing genetic variation could translate into different rates or trajectories of flower-color evolution or diversification in bird vs. bee-pollinated lineages. Interestingly, diversification rates can be often higher in bird-pollinated than bee-pollinated lineages (e.g., *Aquilegia*, Bastida et al., 2010; Bromeliaceae, Givnish et al., 2014; Gesneriaceae, Serrano-Serrano et al., 2017), but counterexamples exist where the reverse is true (*Penstemon*, Wessinger et al., 2019).

### Future Work

Improving our understanding of the relationship between pollinator-mediated selection and floral color variation will require progress on the mechanics of categorical vision (Caves et al., 2018; Zipple et al., 2019). Unfortunately, at this time the categorical boundaries are not mapped with enough precision to apply them to datasets such as ours; for example, the UV region of the spectrum has not been explored for possible boundaries in birds. Further, it is unknown how widespread the phenomenon is among birds beyond zebra finches. Hopefully with progress on mapping boundaries with visual spaces, we could determine if intrapopulation floral color variation tends to span (or not span) category boundaries and thus infer whether selective discrimination between particular floral color variants is even possible.

Further comparative analyses of patterns of floral color variation in different groups are needed to determine how often floral color appears to be constrained within pollinator visual spaces. For example, comparisons of bee- vs. fly-pollinated plant species should be informative as they would be freer of the noise associated with the vertebrate vs. invertebrate sensory and cognitive differences discussed above.

Finally, it would be beneficial to more explicitly tie current selection to floral color variation. Field studies in wild populations could test whether different pollinator groups impose different amounts of stabilizing selection on flower color via inclusion of quadratic terms in phenotypic selection analyses (Lande and Arnold, 1983). Further, experimental evolution approaches could be used to explicitly document the rate of loss of floral color variation from artificially constructed, high-variance plant populations when exposed to different pollinators. While focused on a different question, the feasibility of this approach has been demonstrated by Gervasi and Schiestl (2017), who found rapid divergence of floral characters in experimental populations of fast cycling *Brassica rapa* exposed to bee vs. hoverfly pollinators.

## Data Availability Statement

The dataset generated and analyzed during the current study is available from the Open Science Framework repository at https://doi.org/10.17605/OSF.IO/4B7ES.

## Author Contributions

KW conceived the idea and led the writing of the manuscript. AS and KW designed the study, and collected plant samples and the spectral data. AS extracted pollinator classifications from the literature. CW collected samples, performed the plant species identifications, and curated the specimens. KW and TW analyzed the data. All authors contributed critically to the drafts and gave final approval for publication.

## Conflict of Interest

The authors declare that the research was conducted in the absence of any commercial or financial relationships that could be construed as a potential conflict of interest.

## Footnotes

1 soroherbaria.org, downloaded 2020-07-22.
