# Supplementary material for: Birds Perceive More Intraspecific Color Variation in Bird-Pollinated Than Bee-Pollinated Flowers
Source: Front Plant Sci. 2020 Nov 17;11:590347. doi: 10.3389/fpls.2020.590347 (PMC7705070; doi:10.3389/fpls.2020.590347)
Supplement: Supplementary file 1 [file Data_Sheet_1.pdf]

Appendix Figure S1

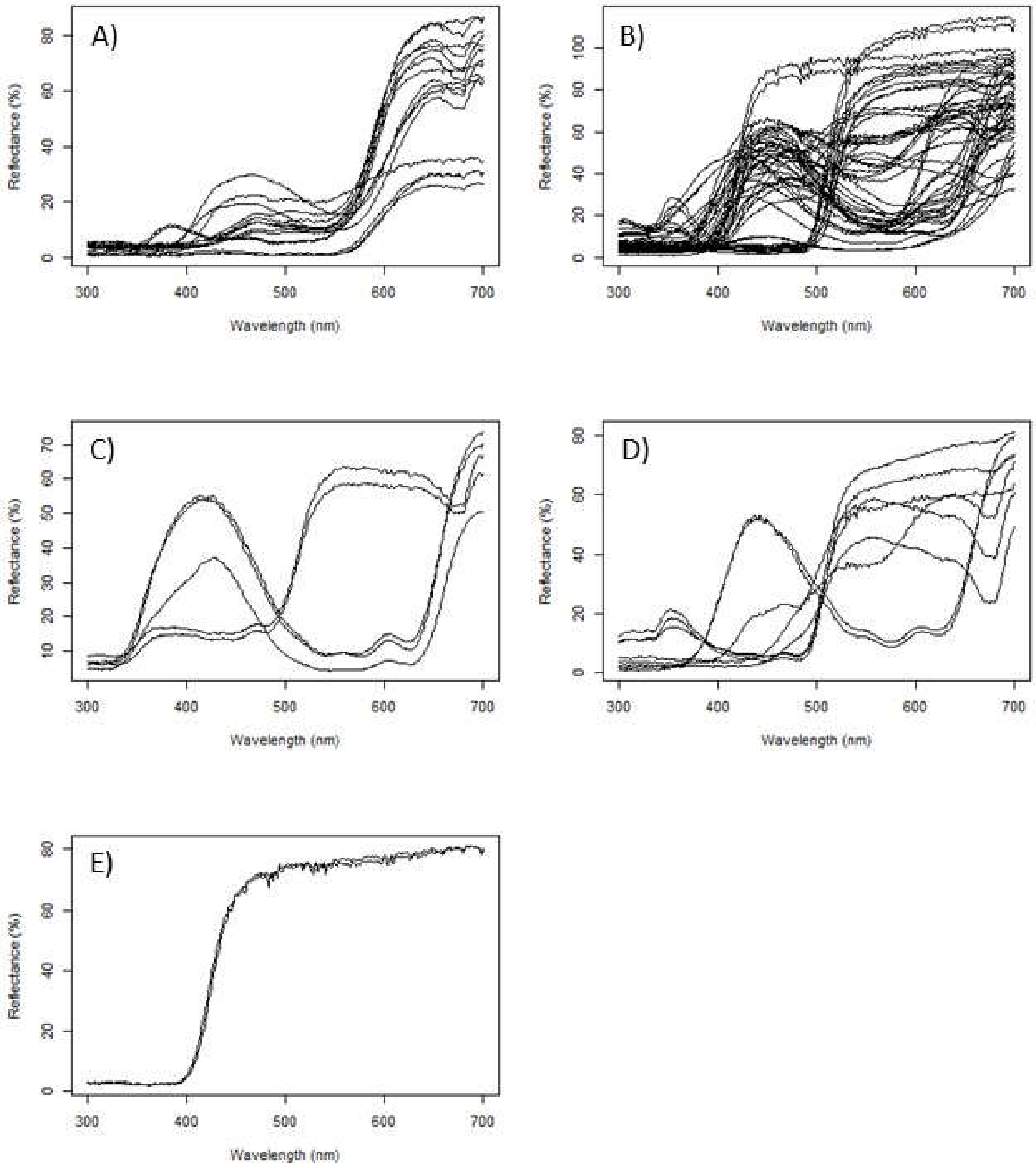

Appendix Fig. S1. Floral spectra for 38 Rocky Mountain plant species grouped by pollination system. Each line is the average spectrum across ~15 individuals within a population; each species is represented by 1-3 populations. A) Seven “Bird” species; B) 24 “Bee” species; C) Two “Bee/Bird” species; D) Four “Bee/Fly” species; E) the single “Bee/Hawkmoth” species.

Appendix Figure S2

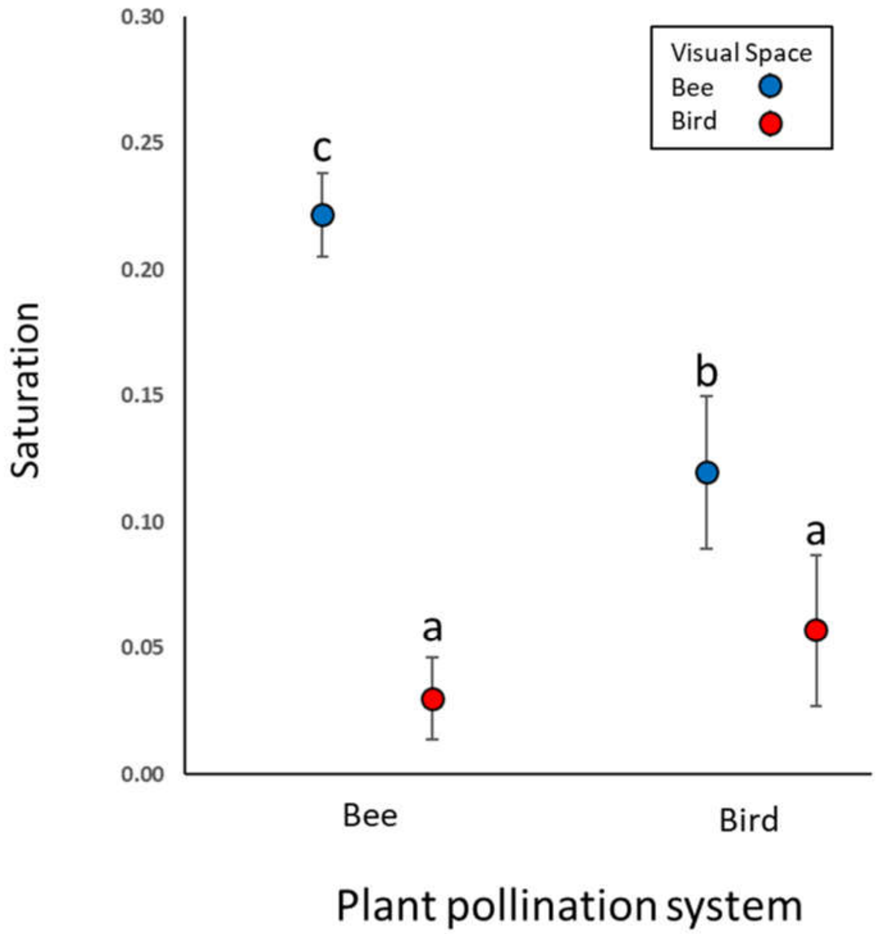

Appendix Fig. S2. Mean saturation (Euclidean distance from the achromatic origin of a visual space) of flowers in bee and bird visual spaces. Estimated model means (with 95% CI) for for 62 populations of 31 Rocky Mountain plant species, grouped by pollination system (24 bee-pollinated and seven bird-pollinated species), are shown. Bars not sharing a letter differ significantly at $p < 0.05$.

Appendix Figure S3

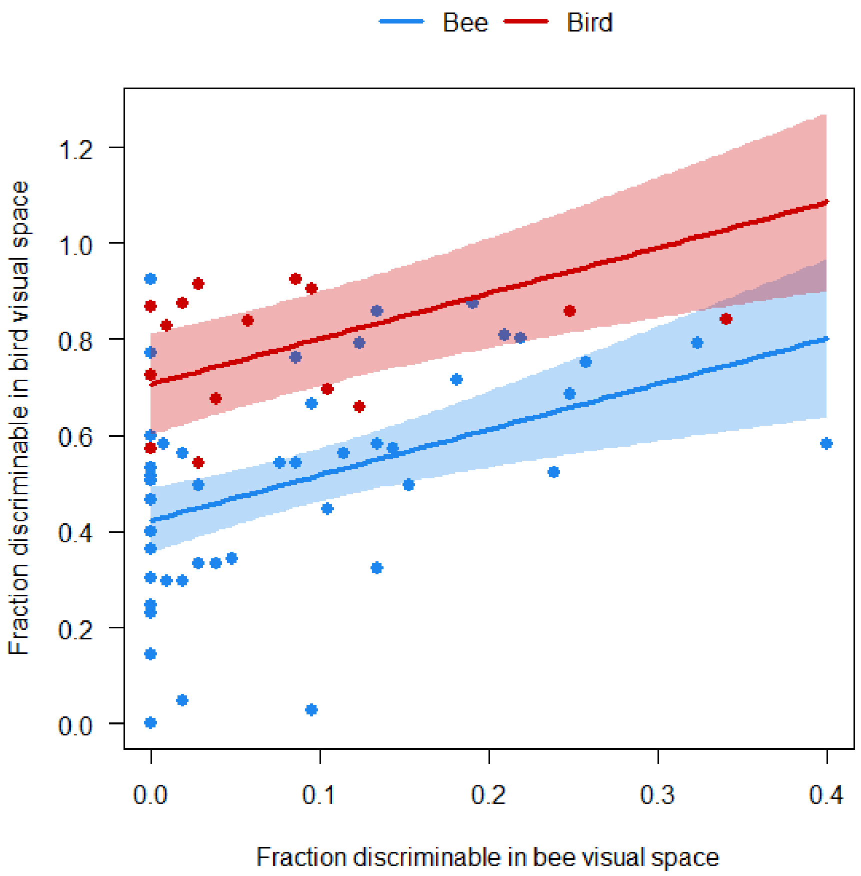

Appendix Figure S3. Variation perceived in bird visual space is positively correlated with that perceived in bee visual space ($p = 0.0003$, adj. $r^2 = 0.38$). Each point represents a plant population (n = 62 populations from 31 species). Axes depict the fraction of intrapopulation flower-flower comparisons that are discriminable to each viewer. Red = plant species with bird pollination system; blue = plant species with bee pollination system. Best-fit lines are plotted with a common slope, as slopes did not differ by pollination system.

Appendix Table S1. Collection and locality information for population-level floral color samples. All sample sizes are n = 15 flowers except for AKS307 (n = 23) and AKS298 (n = 76). All collection dates are in 2019.

Species	Population ID	Date	Latitude	Longitude	Elevation (m)	Location
Aquilegia coerulea	AKS352	7/23	38.9248	-106.9666	2968	Snodgrass
Aquilegia coerulea	AKS389	7/30	38.9645	-106.9937	2914	CR 317 Above Gothic
Aquilegia elegantula	AKS103	7/2	38.9816	-106.9998	2967	Avery Campground
Aquilegia elegantula	AKS283	7/17	38.9579	-106.9869	2896	Research Meadow
Balsamorhiza sagittata	AKS030	6/5	38.6557	-106.8620	2506	Almont Weather Station
Balsamorhiza sagittata	AKS059	6/8	38.7242	-106.8157	2691	Almont Triangle
Campanula rotundifolia	AKS369	7/25	38.8623	-107.0464	2888	Kebler Pass Road
Campanula rotundifolia	AKS382	7/26	38.9575	-106.9852	2917	Warming Meadow
Castilleja chromosa	AKS015	6/3	38.7317	-106.8256	2589	Almont Triangle
Castilleja chromosa	AKS037	6/5	38.6557	-106.8620	2506	Almont Weather Station
Castilleja chromosa	AKS048	6/6	38.7096	-106.8017	2505	Five Mile, Almont
Castilleja linariifolia	AKS096	6/28	38.8865	-106.9681	2752	Mt CB Trail
Castilleja linariifolia	AKS113	7/8	38.8232	-106.8566	2695	Cement Creek Road
Castilleja miniata	AKS392	7/30	38.9750	-106.9970	2923	CR 317 Above Gothic
Castilleja miniata	AKS404	8/5	38.9595	-106.9814	2974	Judd Falls
Castilleja rhexiifolia	AKS393	7/31	38.9774	-106.9747	3481	VA Basin
Castilleja rhexiifolia	AKS401	8/4	39.0182	107.0429	3381	Belleview
Castilleja sulphurea	AKS300	7/18	38.9584	106.9889	2890	Kettle Ponds
Castilleja sulphurea	AKS390	7/30	38.9645	-106.9937	2914	CR 317 Above Gothic
Chamerion angustifolium	AKS402	8/5	38.9580	-106.9890	2887	Road to Gothic
Chamerion angustifolium	AKS405	8/5	38.9595	-106.9814	2974	Judd Falls
Claytonia lanceolata	AKS022	6/4	38.9333	-106.9698	2907	Rosy Point
Claytonia lanceolata	AKS054	6/7	38.9605	-106.9843	2964	Gothic to Judd
Delphinium barbeyi	AKS305	7/18	38.9482	-106.9804	2900	Kettle Ponds
Delphinium barbeyi	AKS351	7/23	38.9248	-106.9666	2968	Snodgrass
Delphinium nuttallianum	AKS003	6/2	38.8594	-106.9251	2772	Upper Loop Trail, jct. Brush Creek Road
Delphinium nuttallianum	AKS006	6/3	38.7226	-106.8182	2675	Almont Triangle
Delphinium nuttallianum	AKS083	6/18	38.8865	-106.9681	2752	Mt CB Trail
Eriogonum umbellatum var. *aureum*	AKS106	7/8	38.6560	-106.8627	2509	Almont Weather Station
Eriogonum umbellatum var. *aureum*	AKS388	7/30	38.9626	-106.9918	2914	CR 317 Above Gothic
Erythronium grandiflorum	AKS052	6/6	38.9204	-106.9618	2933	Start of road to Gothic
Erythronium grandiflorum	AKS153	7/10	38.8501	-107.1003	3043	Kebler Pass
Fragaria virginiana ssp. *glauca*	AKS104	7/2	38.9816	-106.9998	2967	Avery Campground
Fragaria virginiana ssp. *glauca*	AKS239	7/15	38.9767	-106.9773	3429	VA Basin
Frasera speciosa	AKS140	7/10	38.9580	-106.9890	2887	Gothic Townsite
Frasera speciosa	AKS301	7/18	38.9584	106.9889	2890	Kettle Ponds
Geranium viscosissimum	AKS084	6/18	38.8865	-106.9681	2752	Mt CB Trail
Geranium viscosissimum	AKS117	7/9	38.7322	-106.8292	2586	Almont Triangle
Helianthella quinquenervis	AKS306	7/18	38.9482	-106.9804	2900	Kettle Ponds
Helianthella quinquenervis	AKS349	7/23	38.9259	-106.9687	2982	Snodgrass
Hydrophyllum fendleri	AKS211	7/15	38.9950	-107.0042	3053	Rustler's Gulch
Hydrophyllum fendleri	AKS347	7/23	38.9259	-106.9687	2982	Snodgrass
Ipomopsis aggregata	AKS112	7/8	38.8232	-106.8566	2695	Cement Creek Road
Ipomopsis aggregata	AKS186	7/12	38.8697	-107.0177	2815	Kebler Pass Road
Ipomopsis aggregata	AKS353	7/23	38.9227	-106.9648	2960	Snodgrass

Ipomopsis tenuituba	AKS307	7/18	38.8565	-106.7074	3024	Spring Creek, Mosca Campground
Lathyrus lanszwertii	AKS087	6/18	38.8865	-106.9681	2752	Mt CB Trail
Lathyrus lanszwertii	AKS158	7/10	38.8501	-107.1003	3043	Kebler Pass
Linum lewisii	AKS350	7/23	38.9259	-106.9687	2982	Snodgrass
Linum lewisii	AKS381	7/26	38.9575	-106.9852	2917	Warming Meadow
Lonicera involucrata	AKS355	7/24	38.9600	106.9934	2911	Beaver Ponds
Lonicera involucrata	AKS366	7/25	38.8543	-107.0793	2970	Kebler Pass Road
Lupinus polyphyllus	AKS097	6/28	38.8865	-106.9681	2752	Mt CB Trail
Lupinus polyphyllus	AKS298	7/17	38.9547	-106.9878	2883	South Gothic
Mertensia ciliata	AKS303	7/18	38.9479	-106.9734	2888	Kettle Ponds
Mertensia ciliata	AKS348	7/23	38.9259	-106.9687	2982	Snodgrass
Mertensia fusiformis	AKS001	6/2	38.8693	-106.9119	2789	Brush Creek Trail
Mertensia fusiformis	AKS005	6/3	38.7226	-106.8182	2675	Almont Triangle
Mertensia fusiformis	AKS056	6/7	38.9595	-106.9814	2974	Judd Falls
Mimulus guttatus	AKS302	7/18	38.9584	106.9889	2890	Kettle Ponds
Mimulus guttatus	AKS354	7/24	38.9600	106.9934	2911	Beaver Ponds
Pedicularis bracteosa	AKS294	7/17	38.9333	-106.9698	2907	Rosy Point
Pedicularis bracteosa	AKS304	7/18	38.9479	-106.9734	2888	Kettle Ponds
Penstemon caespitosus	AKS089	6/19	38.7229	-106.8176	2700	Almont Triangle
Penstemon caespitosus	AKS315	7/19	38.9791	-106.9783	3560	Avery, north face
Penstemon rydbergii	AKS282	7/17	38.9580	-106.9890	2887	Gothic Townsite
Penstemon rydbergii	AKS285	7/17	38.9051	-107.0195	2728	Slate River Road
Penstemon strictus	AKS116	7/9	38.7322	-106.8292	2586	Almont Triangle
Penstemon strictus	AKS206	7/12	38.8704	-106.9999	2768	Kebler Pass Road
Penstemon whippleanus	AKS356	7/25	38.8501	-107.1003	3043	Kebler Pass
Penstemon whippleanus	AKS394	7/31	38.9767	-106.9773	3429	VA Basin
Pentaphylloides floribunda	AKS286	7/17	38.9051	-107.0195	2728	Slate River Road
Pentaphylloides floribunda	AKS299	7/18	38.9584	106.9889	2890	Kettle Ponds
Ribes montigenum	AKS152	7/10	38.8501	-107.1003	3043	Kebler Pass
Vicia americana	AKS447	8/12	38.9580	-106.9890	2887	Gothic Townsite
Viola praemorsa	AKS002	6/2	38.8693	-106.9119	2789	Brush Creek Trail
Viola praemorsa	AKS027	6/4	38.9333	-106.9698	2907	Rosy Point
Viola praemorsa	AKS078	6/13	38.9594	-106.9871	2884	Treasury, Gothic

Appendix Table S2. Categorizations of pollination systems for the plant species examined in this study. When evidence was not available for the focal species, information from congenerics (listed in "Notes" column) was used instead.

Plant species	Pollinator	Evidence Type	Citation	Notes	Pollination system category
Aquilegia coerulea	Bee (*Bombus*)	Pollen deposition	Brunet and Holmquist 2009	Higher outcrossing rate than hawkmoth	Bee/ Hawkmoth
	Bee (*Bombus*)	Visitation	Brunet and Sweet 2006	Not correlated with outcrossing rate	
	Bee (*Bombus*)	Pollen carried	Miller 1978	Most common	
	Fly	Visitation	Brunet and Sweet 2006	Not correlated with outcrossing rate	
	Hawkmoth	Pollen deposition	Brunet and Holmquist 2009		
	Hawkmoth	Visitation	Brunet and Sweet 2006	Visitation correlated with outcrossing	
	Hawkmoth	Pollen carried	Miller 1978	Common	
	Hummingbird	Stigma contact	Miller 1978	Less common	
Aquilegia elegantula	Bee (*Bombus*)	Pollen carried	Miller 1978	Effective	Bird
	Hummingbird	Morphology	Miller and Willard 1983	Smaller nectar spurs favor bird pollination	
	Hummingbird	Stigma contact	Miller 1978	visits before bees, and is likely more effective	
	Hummingbird	Visitation	Waser 1983	moderate	
Balsamorhiza sagittata	Bee (*Osmia*)	Visitation	Cane and Love 2016	Visitation required for seed set	Bee
	Bee (*Osmia*)	Pollen carried, visitation correlated with seed production	Cane 2005	"Bees are undoubtedly the primary pollinators"	
Campanula rotundifolia	Bee (*Bombus* and others)	Pollen deposition	Bingham and Orthner 1998	Bumblebees deposit most pollen	Bee
	Bee (*Bombus*, *Megachile*)	Visitation	Jane Ogilvie, pers. comm.		
Castilleja chromosa	Hummingbird	Morphology + color	Duffield 1972	Red-bracted *Castillejas* are typically bird-pollinated	Bird
Castilleja linariifolia	Hummingbird	Pollen deposition	Caruso 1999		Bird
	Hummingbird	Visitation	Grant and Grant 1966		
Castilleja miniata	Bee (*Bombus*)	Visitation	Herschl and Roy 2007	Less common	Bird
	Fly	Visitation	Herschl and Roy 2007	Less common	
	Hummingbird	Pollen deposition	Caruso 1999		
	Hummingbird	Pollen deposition	Duffield 1972	Bees are too small to pollinate	
	Hummingbird	Visitation	Grant and Grant 1966		
	Hummingbird	Visitation	Herschl and Roy 2007	Most common	
	Hummingbird	Pollen deposition	Waser 1983		
Castilleja rhexiifolia	Bee	Visitation	Thomson 1982	Less common	Bird
	Bee (*Bombus*)	Visitation	Herschl and Roy 2007	Less common	
	Fly	Visitation	Herschl and Roy 2007	Less common	
	Hummingbird	Visitation, hybridization with *C. miniata*	Herschl and Roy 2007	Most common	
	Hummingbird	Visitation	Thomson 1982		

Castilleja sulphurea	Bee	Visitation	Thomson 1982	Bee visitation more common than in other *Castillejas*	Bee
	Bee (*Bombus*)	Visitation	Herschl and Roy 2007	Most common	
	Bee (*Bombus*)	Review	Waser et al. 2017	Primary	
	Fly	Visitation	Herschl and Roy 2007	Less common	
	Hummingbird	Visitation	Herschl and Roy 2007	Less common	
	Hummingbird	Visitation, hybridization with miniata	Thomson 1982		
	Hummingbird	Visitation	Waser 1983	Moderate	
Chamerion angustifolium	Bee (*Bombus*)	Visitation	Buchanan and Underwood 2013		Bee
	Bee (*Bombus*)	Pollen deposition	Galen and Plowright 1985		
	Bee (*Bombus*)	Visitation	Pengelly and Cartar 2011		
	Hummingbird	Visitation	Waser 1983	Moderate	
Claytonia lanceolata	Bee	Pollen deposition	Parker et al. 2016	In *C. virginica*	Bee
	Bee (*Lasioglossum*)	Visitation, correlated with fertilization	Gezon et al. 2016		
	Bee (*Lasioglossum, Andrena*, *Bombus*)	Visitation	Jane Ogilvie, pers. comm.		
	Fly	Visitation, correlated with fertilization	Gezon et al. 2016		
	Fly	Pollen deposition	Parker et al. 2016	In *C. virginica*. Less effective	
	Fly	Visitation	Jane Ogilvie, pers. comm.		
Delphinium barbeyi	Bee (*Bombus*)	Visitation	Elliot and Irwin 2009	"Primary pollinator"	Bee
	Bee (*Bombus*)	Visitation	Williams et al. 2001	Predominantly outcrossing	
	Bee (*Bombus*)	Visitation	Briggs et al. 2015	Only one hummingbird visit in thousands of hours	
	Hummingbird	Visitation	Waser 1983	Common	
	Hummingbird	Visitation	Williams et al. 2001	Predominantly outcrossing	
	Bee, Hummingbird	Visitation	Nickolas Waser pers. comm.	“Very roughly pollinated 50/50 by hummingbirds and bees”	
Delphinium nuttallianum	Bee (*Bombus*, Halictidae)	Visitation	Waser 1983	85% of visits	Bee/Bird
	Bee (*Bombus*)	Visitation correlated with seed set	Waser and Price 1981	More important than hummingbirds at certain times	
	Bee (*Bombus*)	Review	Waser et al. 2017		
	Bee (*Bombus*)	Pollen carried	Waser 1978	Visitation rare	
	Hummingbird	Visitation	Bosch and Waser 1999	12% of visits	
	Hummingbird	Visitation correlated with seed set	Waser and Price 1981	More important than bees at certain times of year	
	Hummingbird	Review	Waser et al. 2017		
	Hummingbird	Pollen carried	Waser 1978	Visitation common	
	Hummingbird	Pollen deposition	Waser 1983	Visitation common	
Eriogonum umbellatum var. *aureum*	Bee	Pollen carried	Tepedino et al. 2011	In *E. pelinophilum*	Bee/Fly

	Bee	Visitation	Jane Ogilvie, pers. comm.		
	Fly	Pollen carried	Tepedino et al. 2011	Slightly higher composite Total Pollination Value. Study on *E. pelinophilum*	
	Fly	Visitation	Jane Ogilvie, pers. comm.		
	Butterfly	Visitation	Jane Ogilvie, pers. comm.		
Erythronium grandiflorum	Bee	Pollen Deposition	Thomson et al. 1986		Bee
	Bee	Pollen Deposition	Thomson 1986		
	Hummingbird	Visitation	Waser 1983	"Rare"	
Fragaria virginiana ssp. *glauca*	Bee	Stigma Contact	Ashman and King 2005		Bee
	Bee	Visitation	Ashman et al. 2009		
	Bee	Visitation	Case and Ashman 2009	More common	
	Fly	Stigma Contact	Ashman and King 2005		
	Fly	Visitation	Case and Ashman 2009	Less common	
Frasera speciosa	Bee (*Bombus* and *Osmia*)	Pollen Deposition	Beattie et al. 1973	Most efficient	Bee
	Bee (Halictidae)	Visitation	Norment 1988		
	Fly	Pollen Deposition	Beattie et al. 1973	Common, carried less pollen	
	Fly	Visitation	Norment 1988	Most common	
	Hummingbird	Visitation	Waser 1983	"Moderate"	
	Moth	Pollen Deposition	David Inouye, pers. comm.		
Geranium viscosissimum	Bee (*Bombus* and *Osmia*)	Visitation + confirmed outcrossing	Williams et al. 2000	In *G. richardsonii*	Bee
	Bee (*Bombus*)	Pollen carried	Green 1978		
	Bee (*Bombus*)	Visitation and Morphology	Waddington 1979		
	Beetle	Pollen carried	Green 1978		
	Fly	Visitation + confirmed outcrossing	Williams et al. 2000	In *G. richardsonii* Less common	
Helianthella quinquenervis	Bee	Visitation	Thomson 1981		Bee
	Bee (*Bombus*)	Pollen carried	Davendonis and Menke 2009	Nearly exclusive visitor	
	Bee (*Bombus*, *Megachile*, *Osmia*)	Visitation	Jane Ogilvie, pers. comm.		
	Fly	Visitation	David Inouye pers. comm.	"Sometimes common"	
	Fly	Visitation	Jane Ogilvie, pers. comm.		
	Hummingbird	Visitation	Paul CaraDonna pers. comm.	Rare	
Hydrophyllum fendleri	Bee (*Apis*)	Visitation	Wolf 1993	In *H. appendiculatum*	Bee
	Bee (*Bombus* and *Osmia*)	Pollen carried	Beckman 1979	Obligate outcrosser	
	Bee (*Bombus*, *Megachile*, *Lasioglossum, Anthophora*)	Visitation	Jane Ogilvie, pers. comm.		
	Hummingbird	Visitation	Waser 1983	"Moderate"	
Ipomopsis aggregata	Bee (*Bombus*)	Pollen carried	Waser 1978	Visitation common	Bird
	Hawkmoth	Pollen deposition	Campbell et al. 1998	Unknown if hawkmoths are effective pollinators	
	Hummingbird	Pollen Deposition	Campbell et al. 1998		

	Hummingbird	Visitation	Grant and Grant 1966		
	Hummingbird	Visitation	Meléndez-Ackerman and Campbell 1998		
	Hummingbird	Pollen Deposition	Price and Waser 1982		
	Hummingbird	Pollen carried	Waser 1978	Visitation common	
	Hummingbird and Bee (*Bombus*)	Seed set	Mayfield et al. 2001	While *Bombus* was more effective on a per-visit basis, higher visitation by birds resulted in 6.4X more seed set attributed to birds than bees	
Ipomopsis tenuituba	Hawkmoth	Pollen deposition	Campbell et al. 1998	Hawkmoths rare in this area	Bird
	Hummingbird	Pollen deposition	Campbell et al. 1998		
	Hummingbird	Visitation	Meléndez-Ackerman and Campbell 1998		
Lathyrus lanszwertii	Bee	Visitation	Forrest et al. 2010	Primary	Bee
	Bee (*Bombus* and *Osmia*)	Visitation	Paul CaraDonna and Jane Ogilvie, pers. comm.		
Linum lewisii	Bee (*Apis*, *Bombus*, Halictid)	Pollen deposition	Kearns and Inouye 1994	More effective at deposition	Bee/Fly
	Bee (Solitary)	Pollen carried	Kearns 1992		
	Bee (*Bombus*, *Megachile*, *Anthophora*)	Visitation	Jane Ogilvie, pers. comm.		
	Fly	Pollen deposition	Kearns and Inouye 1994	More common, and deemed primary pollinator	
	Fly	Pollen carried	Kearns 1992	More important at higher elevation	
	Fly	Review	Waser et al. 2017		
	Fly	Visitation	Jane Ogilvie, pers. comm.		
Lonicera involucrata	Bee (*Bombus*)	Visitation	Paul CaraDonna & Jane Ogilvie, pers. comm.	Very common visitors, appear to be pollinating	Bee/Bird
	Hummingbird	Morphology	Cronk and Ojeda 2008	Speculation	
	Hummingbird	Visitation	Nickolas Waser pers. comm.	"Almost certainly pollinated by hummingbirds"	
	Hummingbird	Visitation	Waser 1983	moderate	
Lupinus polyphyllus	Bee (*Apis* and *Bombus*)	Visitation	Schaal and Leverich 1980	In *L. texensis*	Bee
	Bee (*Bombus*)	Visitation and morphology	Waddington 1979	In *L. argenteus*	
	Bee (*Bombus*, *Osmia*, *Megachile*, *Andrena*)	Visitation	Jane Ogilvie, pers. comm.		
Mertensia ciliata	Bee (*Apis* and *Bombus*)	Stigma contact	Pelton 1961	Most common and most effective	Bee
	Bee (*Bombus* and *Osmia*)	Single visit deposit	Gallagher and Campbell 2020	Most common and most effective	
	Fly	Visitation	Gallagher and Campbell 2020	Did not enter corolla, less effective	
	Fly	Single visit deposit	Gallagher and Campbell 2020	Less common	
	Hummingbird	Visitation	Waser 1983	Moderate	
Mertensia fusiformis	Bee (*Bombus* and Halactid)	Visitation	Lin and Forrest 2019		Bee

	Bee (*Bombus* and *Osmia*)	Visitation	Forrest and Thomson 2010		
	Bee (*Bombus* and *Osmia*)	Seed set after visitation	Forrest et al. 2011		
	Fly	Visitation	Lin and Forrest 2019	Less common	
	Hummingbird	Visitation	Waser 1983	Moderate	
Mimulus guttatus	Bee (*Bombus* and others)	Visitation	MacNair et al. 1989		Bee
	Bee (*Bombus*)	Pollen deposition	Russel et al. 2019	In lab	
Pedicularis bracteosa	Bee (*Bombus*)	Pollen carried	Aluri and Robart 1991		Bee
	Bee (*Bombus*)	Pollen carried	Macior 1996	Primary	
	Hummingbird	Visitation	Waser 1983	Moderate	
Penstemon caespitosus	Bee (*Anthophora* and *Osmia*)	Pollen carried	Tepedino et al. 2011b	In *P. caryi*	Bee
	Bee (*Bombus* and *Osmia*)	Pollen carried	Tepedino et al. 1999	In *P. penlandii*	
	Fly	Visitatation	Tepedino et al. 1999	In *P. penlandii*. Not very effective pollinators	
Penstemon rydbergii	Bee	Pollen carried	Tepedino et al. 1999	In P. penlandii	Bee
	Bee (*Anthophora* and *Osmia*)	Pollen carried	Tepedino et al. 2011b	In *P. caryi*	
	Bee (*Osmia* and others)	Pollen carried	Lawson et al. 1989	In *P. haydenii*. Highly effective	
	Bee (*Osmia*)	Visitation	Asher Smith pers. comm.		
	Fly	Visitation	Lawson et al. 1989	In *P. haydenii*. Not effective	
Penstemon strictus	Bee	Pollen Deposition	Castellanos et al. 2003		Bee
	Bee (*Bombus*)	Single visit deposit	Morse et al. 2017	Most effective	
	Bee (*Bombus*)	Visitation	Zung et al. 2015	Most common	
	Hummingbird	Pollen Deposition	Castellanos et al. 2003	Less effective at pollen transfer	
	Hummingbird	Visitation	Nickolas Waser pers. comm.		
	Hummingbird	Visitation	Zung et al. 2015		
Penstemon whippleanus	Bee	Pollen deposition	Castellanos et al. 2003	In *P. strictus*	Bee
	Bee	Visitation	Clements and Long 1923	As *P. glauca*	
	Fly	Visitation	Clements and Long 1923	As *P. glauca*	
Pentaphylloides floribunda	Bee (*Bombus* and others)	Visitation	Ricker et al. 2019	Bees made up the majority of visits	Bee
	Fly	Visitation	Ricker et al. 2019	Relatively small percentage	
	Wasp	Visitation	Ricker et al. 2019	Almost half of visits	
	Lepidoptera	Visitation	Ricker et al. 2019	Relatively small percentage	
Ribes montigenum	Bee	Visitation	Paget-Seekins 2012	Occasional	Bee/Fly
	Fly	Visitation	Paget-Seekins 2012	More common	
	Bee (*Bombus*)	Visitation	Jane Ogilvie, pers. comm.	Occasional	
Vicia americana	Bee (*Bombus*)	Visitation	Asher Smith pers. comm.		Bee
	Bee (*Bombus*)	Pollen carried	Teper 2004	In *V. sativa*. In Europe.	
	Bee (*Bombus*)	Pollen carried	Warakomska and Anasiewicz 1991	In *V. villosa* and *V. sativa*. In Europe.	
	Bee (*Bombus*, *Megachile*, *Osmia*)	Visitation	Jane Ogilvie, pers. comm.		
Viola praemorsa	Bee (*Bombus* and Solitary)	Visitation	Forrest and Thomson 2008		Bee/Fly

	Bee (*Osmia* and *Bombus*)	Visitation	Jane Ogilvie, pers. comm.		
	Fly	Visitation	Forrest and Thomson 2008		
	Fly	Visitation	Jones et al. 2013	Visitation rare, selfing common	

References for Appendix Table S2

Aluri, R. J., and Robart, B. W. (1991). Pollination ecology and endemic trends in *Pedicularis bracteosa* var. *atrosanguinea* Pennell & Thompson (Scrophulariaceae) in North America. *Plant Species Biology* 6, 95–104.

Ashman, T.-L., Bradburn, M., Cole, D. H., Blaney, B. H., and Raguso, R. A. (2005). The scent of a male: The role of floral volatiles in pollination of a gender dimorphic plant. *Ecology* 86, 2099–2105. doi:10.1890/04-1161.

Ashman, T.-L., and King, E. A. (2005). Are flower-visiting ants mutualists or antagonists? A study in a gynodioecious wild strawberry. *Am. J. Bot.* 92, 891–895. doi:10.3732/ajb.92.5.891.

Beattie, A. J., Breedlove, D. E., and Ehrlich, P. R. (1973). The ecology of the pollinators and predators of *Frasera Speciosa*. *Ecology* 54, 81–91. doi:10.2307/1934376.

Beckmann, R. L. (1979). Biosystematics of the genus *Hydrophyllum* L. (Hydrophyllaceae). *American Journal of Botany* 66, 1053–1061.

Bingham, R. A., and Orthner, A. R. (1998). Efficient pollination of alpine plants. *Nature* 391, 238–239. doi:10.1038/34564.

Bosch, M., and Waser, N. M. (1999). Effects of local density on pollination and reproduction in *Delphinium nuttallianum* and *Aconitum columbianum* (Ranunculaceae). *Am. J. Bot.* 86, 871–879. doi:10.2307/2656707.

Briggs, H. M., Anderson, L. M., Atalla, L. M., Delva, A. M., Dobbs, E. K., and Brosi, B. J. (2015). Heterospecific pollen deposition in *Delphinium barbeyi*: Linking stigmatic pollen loads to reproductive output in the field. *Ann Bot* 117, 341–247. doi:10.1093/aob/mcv175.

Brunet, J., and Holmquist, K. G. A. (2009). The influence of distinct pollinators on female and male reproductive success in the Rocky Mountain columbine. *Molecular Ecology* 18, 3745–3758. doi:10.1111/j.1365-294X.2009.04304.x.

Brunet, J., and Sweet, H. R. (2006). Impact of insect pollinator group and floral display size on outcrossing rate. *Evolution* 60, 234–246. doi:10.1111/j.0014-3820.2006.tb01102.x.

Buchanan, A. L., and Underwood, N. (2013). Attracting pollinators and avoiding herbivores: insects influence plant traits within and across years. *Oecologia* 173, 473–482. doi:10.1007/s00442-013-2629-4.

Campbell, D. R., Waser, N. M., and Wolf, P. G. (1998). Pollen transfer by natural hybrids and parental species in an *Ipomopsis* hybrid zone. *Evolution* 52, 1602–1611. doi:10.1111/j.1558-5646.1998.tb02241.x.

Cane, J. H. (2005). Pollination needs of arrowleaf balsamroot, *Balsamorhiza sagittata* (Heliantheae: Asteraceae). *Western North American Naturalist* 65, 359–364.

Cane, J. H., and Love, B. (2016). Floral guilds of bees in sagebrush steppe: Comparing bee usage of wildflowers available for postfire restoration. *Natural Areas Journal* 36, 377–391. doi:10.3375/043.036.0405.

Caruso, C. M. (1999). Effect of competition for pollination on the ecology and evolution of a hummingbird-pollinated plant, *Ipomopsis aggregata*.

Case, A. L., and Ashman, T.-L. (2009). Resources and pollinators contribute to population sex-ratio bias and pollen limitation in *Fragaria virginiana* (Rosaceae). *Oikos* 118, 1250–1260. doi:10.1111/j.1600-0706.2009.17520.x.

Castellanos, M. C., Wilson, P., and Thomson, J. D. (2003). Pollen transfer by hummingbirds and bumblebees, and the divergence of pollination modes in *Penstemon*. *Evolution* 51, 2742–2752.

Clements, F. E., and Long, F. L. (1923). “5. Pollinators and flowers visited & 6. Flowers and their visitors,” in *Experimental Pollination; an Outline of the Ecology of Flowers and Insects* (Carnegie Institute), 249–260. Available at: https://digitalcommons.usu.edu/bee_lab_ca/169.

Cronk, Q., and Ojeda, I. (2008). Bird-pollinated flowers in an evolutionary and molecular context. *Journal of Experimental Botany* 59, 715–727. doi:10.1093/jxb/ern009.

Davendonis, J. M., and Menke, S. (2009). Protection at a price: ant interactions with pollinators on aspen sunflower (*Helianthella quinquenervis*). Unpublished Report. Rocky Mountain Biological Lab, Gothic, Colorado USA.

Duffield, W. J. (1972). Pollination ecology of *Castilleja* in Mount Rainier National Park. *The Ohio Journal of Science* 72, 110–114.

Elliott, S. E., and Irwin, R. E. (2009). Effects of flowering plant density on pollinator visitation, pollen receipt, and seed production in *Delphinium barbeyi* (Ranunculaceae). *American Journal of Botany* 96, 912–919. doi:10.3732/ajb.0800260.

Forrest, J., Inouye, D. W., and Thomson, J. D. (2010). Flowering phenology in subalpine meadows: does climate variation influence community co-flowering patterns? *Ecology* 91, 431–440. doi:10.1890/09-0099.1.

Forrest, J. R. K., Ogilvie, J. E., Gorischek, A. M., and Thomson, J. D. (2011). Seasonal change in a pollinator community and the maintenance of style length variation in *Mertensia fusiformis* (Boraginaceae). *Annals of Botany* 108, 1–12. doi:10.1093/aob/mcr093.

Forrest, J., and Thomson, J. D. (2008). Pollen limitation and cleistogamy in subalpine *Viola praemorsa*. *Botany* 86, 511–519. doi:10.1139/B08-020.

Forrest, J., and Thomson, J. D. (2010). Consequences of variation in flowering time within and among individuals of *Mertensia fusiformis* (Boraginaceae), an early spring wildflower. *American Journal of Botany* 97, 38–48. doi:10.3732/ajb.0900083.

Galen, C., and Plowright, R. C. (1985). Contrasting movement patterns of nectar-collecting and pollen-collecting bumble bees (*Bombus terricola*) on fireweed (*Chamaenerion angustifolium*) inflorescences. *Ecological Entomology* 10, 9–17. doi:10.1111/j.1365-2311.1985.tb00530.x.

Gallagher, M. K., and Campbell, D. R. (2020). Pollinator visitation rate and effectiveness vary with flowering phenology. *Am J Bot* 107, 445–455. doi:10.1002/ajb2.1439.

Gezon, Z. J., Inouye, D. W., and Irwin, R. E. (2016). Phenological change in a spring ephemeral: Implications for pollination and plant reproduction. *Glob Change Biol* 22, 1779–1793. doi:10.1111/gcb.13209.

Grant, V., and Grant, K. (1966). Records of hummingbird pollination in the Western American flora. *Aliso: A Journal of Sysematic and Evolutionary Botany* 6, 51–66.

Green, B. B. (1978). Comparative ecology of *Geranium richardsonii* and *Geranium nervosum*. *Bulletin of the Torrey Botanical Club* 105, 108–133. doi:10.2307/2484428.

Herschl, E. I., and Roy, B. A. (2007). Context-dependent pollinator behavior: An explanation for patterns of hybridization among three species of Indian paintbrush. *Evolution* 61, 111–124. doi:10.1111/j.1558-5646.2007.00009.x.

Jones, N. T., Husband, B. C., and MacDougall, A. S. (2013). Reproductive system of a mixed-mating plant responds to climate perturbation by increased selfing. *Proc. R. Soc. B-Biol. Sci.* 280, 81–91. doi:10.1098/rspb.2013.1336.

Kearns, C. A. (1992). Anthophilous fly distribution across an elevation gradient. *American Midland Naturalist* 127, 172–182. doi:10.2307/2426332.

Kearns, C. A., and Inouye, D. W. (1994). Fly pollination of *Linum lewisii* (Linaceae). *American Journal of Botany* 81, 1091–1095. doi:10.1002/j.1537-2197.1994.tb15602.x.

Lawson, H. R., Tepedino, V. J., and Griswold, T. L. (1989). Pollen collectors and other insect visitors to *Penstemon haydenii* S. Wats. *Proceedings of the North American Prairie Conferences* 30, 233–235.

Lin, S.-Y., and Forrest, J. R. K. (2019). The function of floral orientation in bluebells: interactions with pollinators and rain in two species of *Mertensia* (Boraginaceae). *Journal of Plant Ecology* 12, 113–123. doi:10.1093/jpe/rtx073.

Macior, L. W. (1996). Pollination ecology of *Pedicularis bracteosa* in the montane-subalpine ecotone. *Plant Species Biology* 11, 165–171.

Macnair, M. R., Macnair, V. E., and Martin, B. E. (1989). Adaptive speciation in *Mimulus*: an ecological comparison of *M. cupriphilus* with its presumed progenitor, *M. guttatus*. *New Phytol* 112, 269–279. doi:10.1111/j.1469-8137.1989.tb02383.x.

Mayfield, M. M., Waser, N. M., and Price, M. V. (2001). Exploring the "most effective pollinator principle" with complex flowers: Bumblebees and *Ipomopsis aggregata*. *Annals of Botany* 88, 591–596. doi:10.1006/anbo.2001.1500.

Meléndez-Ackerman, E., and Campbell, D. R. (1998). Adaptive significance of flower color and inter-trait correlations in an *Ipomopsis* hybrid zone. *Evolution* 52, 1293–1303.

Miller, R. B. (1978). The pollination ecology of *Aquilegia elegantula* and *A. caerulea* (Ranunculaceae) in Colorado. *American Journal of Botany* 65, 406–414.

Miller, R. B., and Willard, C. L. (1983). The pollination ecology of *Aquilegia micrantha* (Ranunculaceae) in Colorado. *The Southwestern Naturalist* 28, 157–164. doi:10.2307/3671384.

Morse, A., Recart, W., and Campbell, D. (2017). Variability in the effectiveness of *Penstemon strictus* pollinators and the role that water availability plays. Unpublished Report. Rocky Mountain Biological Lab, Gothic, Colorado USA.

Norment, C. J. (1988). The effect of nectar-thieving ants on the reproductive success of *Frasera speciosa* (Gentianaceae). *American Midland Naturalist* 120, 331–336. doi:10.2307/2426005.

Paget-Seekins, J. (2012). *Ribes* (Grossulariaceae) pollination in northern California: Strong overlap in visitor assemblages despite floral diversity.

Parker, A. J., Williams, N. M., and Thomson, J. D. (2016). Specialist pollinators deplete pollen in the spring ephemeral wildflower *Claytonia virginica*. *Ecol Evol* 6, 5169–5177. doi:10.1002/ece3.2252.

Pelton, J. (1961). An investigation of the ecology of *Mertensia ciliata* in Colorado. *Ecology* 42, 38–52. doi:10.2307/1933266.

Pengelly, C. J., and Cartar, R. V. (2011). Effect of boreal forest logging on nectar production of four understory herbs. *Forest Ecology and Management* 261, 2068–2074. doi:10.1016/j.foreco.2011.02.032.

Price, M. V., and Waser, N. M. (1982). Experimental studies of pollen carryover: Hummingbirds and *Ipomopsis aggregata*. *Oecologia* 54, 353–358. doi:10.1007/BF00380004.

Ricker, J. G., Lubell, J. D., and Brand, M. H. (2019). Comparing insect pollinator visitation for six native shrub species and their cultivars. *horts* 54, 2086–2090. doi:10.21273/HORTSCI14375-19.

Schaal, B. A., and Leverich, W. J. (1980). Pollination and banner markings in *Lupinus texensis* (Leguminosae). *The Southwestern Naturalist* 25, 280–282. doi:10.2307/3671260.

Tepedino, V. J., Bowlin, W. R., and Griswold, T. L. (2011a). Diversity and pollination value of insects visiting the flowers of a rare buckwheat (*Eriogonum pelinophilum*: Polygonaceae) in disturbed and "natural" areas. *Journal of Pollination Ecology* 4, 57–67.

Tepedino, V. J., Griswold, T. L., Freilich, J. E., and Shephard, P. (2011b). Specialist and generalist bee visitors of an endemic beardtongue (*Penstemon caryi:* Plantaginaceae) of the Big Horn Mountains, Wyoming. *Western North American Naturalist* 71, 523–528. doi:10.3398/064.071.0410.

Tepedino, V. J., Sipes, S. D., and Griswold, T. L. (1999). The reproductive biology and effective pollinators of the endangered beardtongue *Penstemon penlandii* (Scrophulariaceae). *Pl Syst Evol* 219, 39–54. doi:10.1007/BF01090298.

Teper, D. (2004). Food plants of *Bombus terrestris* L. determined by palynological analysis of pollen loads. *Journal of Apicultural Science* 48, 75–81.

Thomson, J. D. (1981). Field measures of flower constancy in bumblebees. *American Midland Naturalist* 105, 377–380. doi:10.2307/2424756.

Thomson, J. D. (1982). Patterns of visitation by animal pollinators. *Oikos* 39, 241–250. doi:10.2307/3544491.

Thomson, J. D. (1986). Pollen transport and deposition by bumble bees in *Erythronium*: Influences of floral nectar and bee grooming. *The Journal of Ecology* 74, 329–341. doi:10.2307/2260258.

Thomson, J. D., Price, M. V., Waser, N. M., and Stratton, D. A. (1986). Comparative studies of pollen and fluorescent dye transport by bumble bees visiting *Erythronium grandiflorum*. *Oecologia* 69, 561–566. doi:10.1007/BF00410363.

Waddington, K. D. (1979). Divergence in inflorescence height: An evolutionary response to pollinator fidelity. *Oecologia* 40, 43–50. doi:10.1007/BF00388809.

Warakomska, Z., and Anasiewicz, A. (1991). Pollen food of bumblebees caught on *Vicia villosa* Roth. and *Vicia sativa* L. *Ekologia Polska* 39, 391–402.

Waser, N. M. (1978). Competition for hummingbird pollination and sequential flowering in two Colorado wildflowers. *Ecology* 59, 934–944. doi:10.2307/1938545.

Waser, N. M. (1983). "The adaptive nature of floral traits: ideas and evidence," in *Pollination Biology* (Academic Press), 241–278.

Waser, N. M., and Price, M. V. (1981). Pollinator choice and stabilizing selection for flower color in *Delphinium nelsonii*. *Evolution* 35, 276–390.

Waser, N. M., Price, M. V., Casco, G., Diaz, M., Morales, A. L., and Solverson, J. (2017). Effects of road dust on the pollination and reproduction of wildflowers. *International Journal of Plant Sciences* 178, 85–93. doi:10.1086/689282.

Williams, C. F., Kuchenreuther, M. A., and Drew, A. (2000). Floral dimorphism, pollination, and self-fertilization in gynodioecious *Geranium richardsonii* (Geraniaceae). *Am. J. Bot.* 87, 661–669. doi:10.2307/2656852.

Williams, C. F., Ruvinsky, J., Scott, P. E., and Hews, D. K. (2001). Pollination, breeding system, and genetic structure in two sympatric *Delphinium* (Ranunculaceae) species. *Am. J. Bot.* 88, 1623–1633. doi:10.2307/3558407.

Wolfe, L. M. (1993). Reproductive consequences of a flower color polymorphism in *Hydrophyllum appendiculatum*. *American Midland Naturalist* 129, 405–408. doi:10.2307/2426522.

Zung, J. L., Forrest, J. R. K., Castellanos, M. C., and Thomson, J. D. (2015). Bee- to bird-pollination shifts in *Penstemon*: effects of floral-lip removal and corolla constriction on the preferences of free-foraging bumble bees. *Evol Ecol* 29, 341–354. doi:10.1007/s10682-014-9716-9.

Appendix Table S3. Notes on flower preparation for each plant species prior to spectrometry.

Species	Target and preparation
Aquilegia coerulea	Petal face (white)
Aquilegia elegantula	Side of nectar spur, mounted on tape
Balsamorhiza sagittata	Petal face, quarter of the way from center
Campanula rotundifolia	Outside of corolla, still attached to flower
Castilleja chromosa	Inside of bract, mounted on tape
Castilleja linariifolia	Inside of bract, mounted on tape
Castilleja miniata	Inside of bract, mounted on tape
Castilleja rhexiifolia	Inside of bract, mounted on tape
Castilleja sulphurea	Inside of bract, mounted on tape
Chamerion angustifolium	Lower petal, face
Claytonia lanceolata	Petal face, mounted on tape
Delphinium barbeyi	Lower petal, face
Delphinium nuttallianum	Lower petal, face
Eriogonum umbellatum var. *aureum*	Side of flower, mounted on tape
Erythronium grandiflorum	Mount top petal on tape, shoot widest part
Fragaria virginiana ssp. *glauca*	Petal face
Frasera speciosa	Face of petal near tip, ripped off
Geranium viscosissimum	Petal face
Helianthella quinquenervis	Widest part of random petal
Hydrophyllum fendleri	Tear off petal, inside
Ipomopsis aggregata	Side of floral tube, mounted on tape
Ipomopsis tenuituba	Side of floral tube, mounted on tape
Lathyrus lanszwertii	Bottom of banner
Linum lewisii	Petal face
Lonicera involucrata	Side of tube, mounted on tape
Lupinus polyphyllus	Side of keel, mounted on tape
Mertensia ciliata	Side of flower, mounted on tape
Mertensia fusiformis	Side of flower, mounted on tape
Mimulus guttatus	Inside of lower, outer lobe, mounted on tape
Pedicularis bracteosa	Inside of lower lobe, mounted on tape
Penstemon caespitosus	Side of tube, mounted on tape
Penstemon rydbergii	Inside of one of the bottom lobes, mounted on tape
Penstemon strictus	Inside of one of the bottom lobes, mounted on tape
Penstemon whippleanus	Inside of one of the bottom lobes, mounted on tape
Pentaphylloides floribunda	Petal face
Phlox multiflora	Center of petal face, mounted on tape
Ribes montigenum	Tear off petal, mounted on tape
Veratrum californicum	Petal face, mounted on tape
Vicia americana	Outside of keel, mounted on tape
Viola praemorsa	Middle of bottom petal, mounted on tape

Appendix Table S4. Statistical results for the model associated with Fig. 2, but with alternative species groupings and numbers of species included in the analysis.

Alternative species grouping	No. species	main effect of visual system		main effect of pollination system		visual space x pollination system interaction		Between pollination system Contrast (within bee space)	Between pollination system Contrast (within bird space)
		χ^2	P	χ^2	P	χ^2	P	P	P
Base case	31	488.6	<0.0001	7.4	0.0065	30.2	<0.0001	0.9585	<0.0001
"Bee/Bird" species included with "Bee" species	33	501.7	<0.0001	7.5	0.0063	24.5	<0.0001	0.8603	<0.0001
"Bee/Bird" species included with "Bird" species	33	526.5	<0.0001	5.7	0.0172	30.9	<0.0001	0.7852	<0.0001
"Bee/Fly" and "Bee/ Hawkmoth" species both included with "Bee" species	36	428.3	<0.0001	6.5	0.0107	28.8	<0.0001	0.8639	<0.0001
